# Umsetzung des Arbeitsschutzes während der SARS-CoV-2-Pandemie in Friseursalons

**DOI:** 10.1007/s40664-021-00433-x

**Published:** 2021-06-15

**Authors:** Martina Michaelis, Ulrich Stößel, Johanna Stranzinger, Albert Nienhaus

**Affiliations:** 1 FFAS – Freiburger Forschungsstelle Arbeits- und Sozialmedizin, Bertoldstr. 63, 79098 Freiburg, Deutschland; 2 Abt. Arbeitsmedizin/Gefahrstoffe/Gesundheitswissenschaften, Berufsgenossenschaft für Gesundheitsdienst und Wohlfahrtspflege (BGW), Hamburg, Deutschland

**Keywords:** Adhärenz, Arbeitsschutzstandard, Infektionsschutz, Studie mit verdeckter teilnehmender Beobachtung, Pandemie, Adherence, Occupational health and safety standard, Infection control, Observation study, Pandemic

## Abstract

**Hintergrund:**

Zur Einhaltung (Adhärenz) von Arbeitsschutzvorgaben bestehen in Deutschland wenig empirische Erkenntnisse. Empfehlungen zur Prävention von SARS-CoV-2-Infektionen im Friseurhandwerk wurden im Frühjahr 2020 von der Berufsgenossenschaft für Gesundheitsdienst und Wohlfahrtspflege (BGW) in einem Arbeitsschutzstandard bereitgestellt.

**Fragestellung:**

Inwieweit werden die Inhalte der Empfehlungen in dieser Branche aus Sicht von Friseurkund:innen umgesetzt und eingehalten?

**Material und Methoden:**

Die Erhebung erfolgte als verdeckte teilnehmende Beobachtungsstudie zwischen Anfang Oktober und Mitte Dezember 2020 als nichtsystematische Gelegenheitsstichprobe in 3 Städten. Das standardisierte Erhebungsinstrument umfasste 3 Bereiche: a) Maßnahmen allgemeiner Art, vorwiegend technischer Art, b) im Kontakt mit der Kundin/dem Kunden und c) auf individueller Ebene zur Infektionsprävention. Die Ergebnisse auf Einzel- und Gesamtebene wurden in einem standardisierten Summenindex (0–1) zur SARS-CoV-2-Arbeitsschutzstandard-Adhärenz zusammengefasst. Mittels nonparametrischem Wilcoxon-Test wurden mögliche Unterschiede zwischen den 3 Subindizes geprüft.

**Ergebnisse:**

Der Gesamtindex zur Adhärenz von 162 Beobachtungen beträgt 0,75 (SD: 0,14). Die Werte der beiden Subindizes zur Infektionsprävention im Kontakt mit dem/der Kund:in (z. B. Hinweis auf Verhaltensregeln) und auf individueller Schutzebene (Tragen einer Mund-Nasen-Bedeckung) sind signifikant besser als der zu allgemeinen Maßnahmen (z. B. Händereinigungsmöglichkeit für Kund:innen).

**Diskussion:**

Die beobachtete Adhärenz zur Vermeidung von SARS-CoV-2-Infektionen in Friseurbetrieben ist höher, als dies subjektive Erfahrungen der BGW zur Arbeitsschutzadhärenz nahelegen. Dies könnte auch mit der in der Öffentlichkeit diskutierten Infektionsgefährdung erklärt werden. Die Ergebnisse weisen eine leicht niedrigere Adhärenz im Vergleich mit Erkenntnissen auf, die in rund 400 standardisierten Befragungen durch die BGW-Präventionsdienste gewonnen wurden.

**Limitationen:**

Angesichts der nichtsystematischen Gelegenheitsstichprobe kann eine (positive) Verzerrung der Ergebnisse nicht ausgeschlossen werden.

## Abstract (en)

**Background:**

There is little empirical evidence with respect to the implementation (adherence) of occupational health and safety guidelines in Germany. Recommendations for the prevention of SARS-CoV‑2 infections in hairdressing services were provided by the German Statutory Accident Insurance for the Health and Welfare Services (BGW) in an occupational health and safety (OHS) standard in spring 2020.

**Research question:**

To what extent are the recommendations adhered to in this service profession as judged by hairdresser’s customers after their visits?

**Material and methods:**

The survey was conducted as a covert participant observational study between the beginning of October and the middle of December 2020 as a non-systematic opportunistic sample in three cities. The standardized checklist included three domains: a) measures of general, mainly technical nature, b) in contact with the customer and c) on an individual level for infection prevention.

The results on domain and overall level were summarized in a standardized sum index (0–1) of SARS-CoV‑2 OHS standard adherence. Nonparametric Wilcoxon tests were used to examine possible differences among the three subindices.

**Results:**

The overall adherence index of 162 observations was 0.75 (SD 0.14). The two subindices on infection prevention in contact with the customer (e.g. indications for behavior rules) and on the individual level (e.g. wearing a mouth-nose covering) were significantly better than the one on general measures (e.g., hand cleaning possibility for customers).

**Discussion:**

The observed adherence for prevention of SARS-CoV-2-infections in hairdresser salons was higher than experiences of the BGW on OHS adherence suggest. This might be explained by the general public awareness of risks of infection. The results on the adherence are only slightly lower than those resulting from around 400 standardized personal surveys of the BGW prevention services.

**Limitations:**

Given the non-systematic opportunistic sample, a (positive) bias in the adherence results cannot be excluded.

Über die tatsächliche Umsetzung (Adhärenz) von Vorgaben im Arbeitsschutz gibt es in Deutschland wenig belastbare Daten. Im Rahmen der SARS-CoV-2-Pandemie wurde von der Berufsgenossenschaft für Gesundheitsdienst und Wohlfahrtspflege (BGW) ein spezifischer Branchenschutzstandard bereitgestellt. Die Umsetzung der darin formulierten Empfehlungen wurde im Rahmen einer Studie mit verdeckter teilnehmender Beobachtung durch Friseurkund:innen festgehalten.

Wie hoch die Einhaltung (Adhärenz) von Arbeitsschutzvorgaben in Deutschland tatsächlich ist, ist empirisch bislang nur wenig untersucht. Defizite werden insbesondere in Klein- und Kleinstbetrieben gefunden [1–3]. In Friseurbetrieben beispielsweise werden nach Erfahrungen der BGW nur etwa 30–60 % der berufsgenossenschaftlichen Arbeitsschutzstandards umgesetzt. Einflussfaktoren sind nicht zuletzt z. B. ihr Bekanntheitsgrad, aber auch innerbetriebliche und individuelle Faktoren, wie u. a. ein Review von Studien aus dem medizinischen Bereich zeigt [4].

Die Adhärenz von Hygieneregeln z. B. für die Hände ist bekanntermaßen seit Jahren sogar in den hinsichtlich nosokomialer Infektionen vulnerablen Einrichtungen der Patientenversorgung defizitär [5, 6], auch wenn sie in Zeiten der SARS-CoV-2-Pandemie vergleichsweise besser ist [7]. Es ist davon auszugehen, dass Betriebe in anderen Branchen mit den in der Pandemie erforderlichen Hygieneanforderungen erst seit ihrem Beginn im Frühjahr 2020 konfrontiert wurden. Im Friseurhandwerk besteht hierzu bereits eine gewisse Affinität durch den Umgang mit Desinfektionsmitteln nach der branchenspezifischen Technischen Regel des Bundesministeriums für Arbeit und Gesundheit [8], der Biostoffverordnung und bundeslandspezifischen Hygieneanforderungen. Sie betreffen die Reinigung und Desinfektion von Arbeitsmaterialien und gewisse Hygienevorgaben für Friseur:innen im Umgang mit Kund:innen.

Um die Übertragung von SARS-CoV‑2 durch Tröpfchen und Aerosole im beruflichen Umfeld zu verhindern, sind die im Arbeitsschutz üblichen technischen, organisatorischen und personenbezogenen Schutzmaßnahmen (TOP-Prinzipien) zu beachten. Für die Zeit nach dem ersten wirtschaftlichen *Lockdown* wurde im Mai 2020 von der BGW der branchenbezogene SARS-CoV-2-Arbeitsschutzstandard für das Friseurhandwerk bereitgestellt [9]. Er wird laufend aktualisiert und konkretisiert bzw. ergänzt Arbeitsschutzmaßnahmen im Sinne einer Richtschnur zur Auslegung des Arbeitsschutzgesetzes. Mittlerweile wurde er mit der „SARS-CoV-2-Arbeitsschutzregel“ und dem „SARS-CoV-2-Arbeitsschutzstandard“ des Bundesministeriums für Arbeit und Soziales [10] abgestimmt. Der Standard für Friseur:innen in der Fassung vom 30.04.2020 beinhaltete unter anderem:

- eine Anpassung der einzelnen Friseurarbeitsplätze auf eine Distanz von mindestens 1,5 m und das konsequente Einhalten des Mindestabstands zwischen allen Personen im Salon außer bei der Haarbehandlung,
- eine ausreichende Belüftung der Räumlichkeiten,
- die Markierung und/oder Absperrung einzelner Bewegungsräume,
- das Aufstellen eines Schutzschilds zwischen Kundschaft und Kasse und das Angebot kontaktlosen Bezahlens,
- den Verzicht auf eine Bewirtung mit Getränken und die Zurverfügungstellung von Zeitschriften nur unter Hygieneauflagen,
- das konsequente Einhalten von Schutzmaßnahmen (medizinischer Mund-Nasen-Schutz, Atemschutz-(FFP2-)Maske oder stoffliche Mund-Nasen-Bedeckung, im Folgenden Mund-Nasen-Bedeckung genannt) bei Friseur:innen und Kund:innen bei Unterschreitung der Mindestabstände, Friseurumhang),
- die Bereitstellung eines Händedesinfektionsmittels und/oder hautschonender Flüssigseife mit Einmalhandtüchern und die dringende Bitte an die Kund:innen, diese zu nutzen,
- die Verwendung von Einmalhandschuhen beim Kontakt mit Haaren und Gesicht mindestens bis nach dem Haarewaschen, das möglichst immer erfolgen sollte, und
- das gleichzeitige Bedienen mehrerer Kund:innen von einer Friseurfachkraft nur unter konsequenter Beachtung von Hygiene und Infektionsschutzmaßnahmen.

In der Fassung vom April 2020 wurde der Verzicht auf gesichtsnahe Dienstleistungen wie Augenbrauen- und Wimpernfärben, Rasieren und Bartpflege empfohlen. Später waren diese Handlungen unter Einsatz einer FFP2-Maske wieder freigegeben; die Maske soll nun zusätzlich mit einer Schutzbrille oder einem Gesichtsschild zum Schutz vor Tröpfcheninfektionen ergänzt werden.

Um einen Überblick über die Einhaltung von Arbeitsschutzregeln im Zusammenhang mit dem Infektionsschutz während der SARS-CoV-2-Pandemie am Beispiel von Friseursalons zu erhalten, konzipierten wir mit finanzieller Unterstützung der BGW eine verdeckte teilnehmende Beobachtungsstudie mit *normalen* Friseurkund:innen. Die Methode wurde gewählt, da bei Selbstangaben der Friseur:innen zu diesem Thema Antworten im Sinne einer sog. sozialen Erwünschtheit zu erwarten gewesen wären [11].

Aus der Sicht der Friseurkund:innen sollte geklärt werden, inwieweit im Friseursalon empfohlene Maßnahmen zum Schutz vor einer SARS-CoV-2-Infektion in den Bereichen a) allgemeiner Art, b) im Kontakt mit den Kund:innen und c) auf individueller Ebene eingehalten werden.

## Methoden

In Anlehnung an Mystery-Research-Methoden [12] wurde jeweils eine verdeckt teilnehmende Fremd-(und Selbst‑)Beobachtung durch Friseurkund:innen durchgeführt. Der Zugang erfolgte im Rahmen einer nichtsystematischen Gelegenheitsstichprobe in 3 Städten. Angestrebt waren ursprünglich jeweils 60 Beobachtungen in Hamburg und Freiburg. Im Studienverlauf wurde die Rekrutierung auf die Stadt Berlin ausgeweitet. Die Beobachtenden wurden im persönlichen und beruflichen Umfeld der Autor:innen und derer Bekannten im Schneeballsystem rekrutiert. In einer schriftlichen Studieninformation wurde auf das verdeckte Vorgehen und das nachträgliche Ausfüllen des Erhebungsinstruments hingewiesen. Der Erhebungszeitraum war zwischen Anfang Oktober und Ende Dezember 2020.

Das Erhebungsinstrument umfasste 18 standardisierte Hauptfragen zu den oben genannten 3 Bereichen und wurde vor dem Einsatz einem Pretest mit 5 Personen unterzogen.

Die ausgefüllten Beobachtungsbögen wurden vor der Weitergabe mit einem Code pseudonymisiert (erster Buchstabe des Salon- und des Straßennamens), sodass bei der Datenauswertung keine rückführenden Bezüge zum besuchten Friseurbetrieb hergestellt werden konnten. Mutmaßliche Mehrfachbeurteilungen ein und desselben Friseursalons wurden auf der Basis gleicher Codes von den Analysen ausgeschlossen (*n* = 14). In den Daten wurden auch die Beobachtenden pseudonymisiert.

### Statistische Analysen

Die Daten wurden deskriptiv ausgewertet. Fehlende Werte wurden von den Berechnungen ausgeschlossen und mögliche regionale Unterschiede explorativ verglichen. Je nach Datenniveau erfolgte für den Städtevergleich ein Chi^2^- oder ein nonparametrischer Mann-Whitney-U-Test mit den dazugehörigen Effektstärken. Bei mehr als 2 Gruppen wurden Post-hoc-Tests berücksichtigt, ggf. mit Bonferroni-adjustierter Signifikanzschwelle zur Reduzierung der Alphafehlerinflation bei Mehrfachtestungen.

Die Ergebnisse der drei genannten Bereiche wurden im zweiten Schritt in jeweils einem standardisierten Summenindex und diese wiederum als standardisierter Gesamt-Mittelwertindex „Adhärenz zum SARS-CoV-2-Arbeitsschutzstandard“ zusammengefasst (Summe der Variablenwerte dividiert durch die Anzahl der Variablen). Der mögliche Wertebereich umfasst 0 und 1 (keine bis bestmögliche Adhärenz). Dabei wurden mehrere Optionen gewichtet (Bereitstellung von Wasser und Seife ohne Desinfektionsmittel = 0,5, Einhaltung der allgemeinen Distanzregel zwischen Kund:innen (meistens = 0,75, selten = 0,25), Verwendung einer Mund-Nasen-Bedeckung, aber nicht konsequent = 0,75).

Mittels nonparametrischem Wilcoxon-Test wurden analog zum obigen Vorgehen mögliche Unterschiede zwischen den 3 Subindizes geprüft. Die zu den jeweiligen Tests dazugehörigen Effektstärken (phi bzw. Kontigenzkoeffizient C beim Chi^2^-Test und r beim Mann-Whitney-U- bzw. Wilcoxon-Test (z/Wurzel[Fallzahl]) wurden mit ≤0,3 (geringer), ≤0,5 (moderater) und ≥0,5 (großer Unterschied) kategorisiert [13]).

## Ergebnisse

### Datenrücklauf/-qualität und Stichprobenmerkmale

Es wurden insgesamt 162 Einmalbeobachtungen in Berlin (*n* = 44), Hamburg (*n* = 65) und Freiburg (*n* = 53) auswertet. Dies entspricht 3 % der in Berlin (von *n* = 1338), 8 % der in Hamburg (*n* = 851) und 43 % der in Freiburg gewerblich angemeldeten Friseursalons (*n* = 124; Recherche laut Branchenverzeichnis von www.dastelefonbuch.de im Dezember 2020). 66,7 % der Beobachtenden waren Frauen (*n* = 108).

Die Ausfüllqualität der Erhebungsbögen war sehr gut bis auf 2 Aspekte; dies betraf Serviceangebote im Sinne von Getränken und/oder Zeitschriften (17,3 % fehlende Angaben) und das Tragen von Einmalhandschuhen bei Haarwäsche/-färbung (11,4 %).

Merkmale des Aufenthalts im Friseursalon und des Anlasses für den Friseurbesuch zeigen Tab. 1 und 2. Für alle Beobachtenden war das Haareschneiden oder -färben der Anlass des Besuchs; weitere gesichtsnahe Tätigkeiten (Rasur, Wimpern- oder Augenbrauenfärben) wurden von 2,5 % in Anspruch genommen (*n* = 4). In 13,0 % der Besuche war die beobachtende Person die einzige Kundschaft im Friseursalon (*n* = 21).

**Table 1:** 

	Prozent	*N*_(ja)_	*N*_(gültig)_
Haareschneiden	96,3	156	162
Haarfärbung	22,2	36	162
Weitere Behandlung(en) ohne Gesichtsnähe (Strähnen, Extension, Dauerwelle)	3,1	5	162
Rasur	0,6	1	162
Wimpernfärbung	1,2	2	162
Augenbrauenfärbung	1,2	2	162

**Table 2:** 

	MW	SD	Median	Spanne	*N*_(gültig)_
Aufenthaltsdauer der Kundin/des Kunden im Friseursalon (h)	1,1	0,8	0,7	0,2–4,0	161
Anzahl der Behandlungsplätze pro qm^2^ ^a^	11,7	7,4	10,0	3,0–54,0	161
Anzahl aller Personen im Friseursalon (gleichzeitig; Kund:innen und Friseur:innen)	5,6	2,6	6,0	2–14	162
Anzahl aller Personen pro qm^2^ ^a^	12,0	7,3	10,0	3,3–60,0	161

Datenbasis: *n* = 162

*MW* Mittelwert, *SD* Standardabweichung

^a^Nach Abschätzung der Beobachtenden

### Einzelne Schutzmaßnahmen

Die meisten Friseur:innen hatten gut sichtbare schriftliche Hinweise auf SARS-CoV-2-Schutzmaßnahmen im Salon angebracht und Händedesinfektionsmittel bereitgestellt (Tab. 3).

**Table 3:** 

	Prozent	*N*_(ja)_	*N*_(gültig)_
*Schriftliche Hinweise auf SARS-CoV-2-Schutzmaßnahmen (gut sichtbar)*	84,0	136	162
*Besondere Lüftung des Raums*^*a*^	61,7	100	162
*Schutzschild zwischen Kasse und Kundschaft*	55,6	90	162
*Bodenmarkierungen als Hinweis zum Abstandhalten*^*b*^	29,6	48	162
*Möglichkeit der Händereinigung für Kund:innen*			162
Händedesinfektionsmittel	83,3	135	
Wasser und Seife	8,0	13	
Keine	8,6	14	
*Einhaltung der allgemeinen Distanzregel zwischen den Kund:innen*^*c*^			162
Immer	80,9	131	
Meistens	18,5	30	
Selten	0,6	1	
Nie	0,0	0	

Datenbasis: *n* = 162

^a^Zum Beispiel ständig geöffnete Außentür, regelmäßige Stoßlüftung

^b^Abstandsmarken, Leitzeichen

^c^Zum Beispiel Abstand zwischen Friseurstühlen oder Waschplätzen, nur jeder zweite Stuhl besetzt, Warten mit räumlichem Abstand etc.

Eine besondere Lüftung des Raums und ein Schutzschild zwischen Kasse und Kundschaft konnten nur etwas mehr als die Hälfte der Beobachtenden feststellen. Bodenmarkierungen als Hinweis zum Abstandhalten vor dem Tresen waren eher selten, allerdings wurde auch jeweils darauf hingewiesen, wenn z. B. nur ein Platz im Salon war und Schutzschild oder Bodenmarkierungen daher nicht notwendig waren.

In den Anmerkungen berichteten die Beobachtenden u. a. eine „besondere Raumlüftung“ (30-mal), permanent geöffnete Türen oder Fenster (20-mal) und Stoßlüften (13-mal). Zweimal wurde explizit der ausschließliche Einsatz einer raumlufttechnischen Anlage genannt und 4‑mal der zusätzliche Einsatz mobiler Luftreiniger mit Aerosolfilter.

Die allgemeine Distanzregel von 1,5 m zwischen Kund:innen konnte von 81 % „immer“ eingehalten werden; war dies nicht der Fall, wurde mehrheitlich auf zu geringe Abstände zwischen den Arbeitsplätzen oder in anderen Bereichen wie Eingang oder Wartebereich hingewiesen (*n* = 9 bzw. 7 von 19 Nennungen).

Eine Begrüßung/Verabschiedung mit z. B. Händeschütteln war nahezu nie der Fall (Tab. 4). Auch wurden die Haare vor der Behandlung fast immer gewaschen, auf ein Angebot von Getränken oder Zeitschriften verzichtet und Kontaktdaten für den Fall einer Nachverfolgung von Infektionsketten erbeten. Seltener (bei rund der Hälfte) wurde bargeldlose Bezahlung angeboten und mündlich auf die SARS-CoV-2-Verhaltensregeln hingewiesen. Zusammen mit den schriftlichen Verhaltensregeln (Tab. 3) informierten 52,2 % der Friseur:innen (*n* = 84) ihre Kund:innen zu den Verhaltensregeln.

**Table 4:** 

	Prozent	*N*_(ja)_	*N*_(gültig)_
Vermeidung einer Begrüßung/Verabschiedung mit körperlichem Kontakt	99,4	161	162
Mündlicher Hinweis auf SARS-CoV-2-Verhaltensregeln^a^	56,8	92	162
Service angeboten (Getränke und/oder Zeitschriften)	4,5	6	134
Händewaschen oder -desinfektion der Kundin/des Kunden, sofern Mittel bereitgestellt (von *n* = 148; Tab. 3)	87,9	130	148
Haarwäsche vor dem Schnitt/der Färbung	81,5	132	162
Angebot einer bargeldlosen Bezahlung	61,1	99	162
Bitte um Hinterlassung von Kontaktdaten für den Fall einer Nachverfolgung von Infektionsketten^b^	87,0	141	162

Datenbasis: *n* = 162

^a^Zum Beispiel Abstand halten, Friseurumhang, Mund-Nasen-Bedeckung, Händehygiene, technische Details

^b^Für den Fall einer Nachverfolgung von Infektionsketten

Einen Mund-Nasen-Schutz trugen fast alle Friseur:innen und alle Kund:innen, wenn auch manche nicht immer vorschriftsmäßig (Tab. 5). Im Fall der wenigen gesichtsnahen Tätigkeiten (*n* = 4) wurde von der Friseurin/vom Friseur eine Mund-Nasen-Bedeckung getragen, nicht aber ein Augen- oder Gesichtsschutz.

**Table 5:** 

	Prozent	*N*_(ja)_	*N*_(gültig)_
*Tragen einer Mund-Nasen-Bedeckung (Friseur:in)*			162
Ja, konsequent	93,2	151	
Ja, aber nicht immer vorschriftsmäßig	6,2	10	
Nein	0,6	1	
*Tragen einer Mund-Nasen-Bedeckung (Kund:in)*			161
Ja, konsequent	94,4	153	
Ja, aber nicht immer vorschriftsmäßig	4,3	7	
Nein	0,0	0	
*Tragen von Einmalhandschuhen bei Haarwäsche/-färbung, sofern erfolgt (von n* *=* *132; Friseur:in)*	62,4	73	117
*Nicht gleichzeitiges Bedienen mehrerer Kund:innen*	87,0	141	162
*Schutzmaßnahmen im Fall gesichtsnaher Tätigkeiten*			4
Mund-Nasen-Bedeckung (Kund:in)	75,0	3	
Augenschutzbrille (Friseur:in)	25,0	1	
Gesichtsschild (Friseur:in)	25,0	1	
Mund-Nasen-Bedeckung (Friseur:in)	100,0	4	

Das Tragen von Einmalhandschuhen bei Haarwäsche/-färbung kann aufgrund eines hohen Anteils fehlender Angaben nicht abschließend beurteilt werden. Würden diese als „Nichttragen“ interpretiert, wäre diesbezüglich eine Arbeitsschutzregel-Adhärenz von rund der Hälfte beobachtet worden (53,0 %). Ein gleichzeitiges Bedienen mehrerer Kund:innen kam nur vereinzelt vor.

### Adhärenz von Schutzstandards als standardisierter Index

Die Adhärenz hinsichtlich Schutzstandards liegt bei insgesamt 75 % (SD: 14 %; siehe Gesamtindex in Tab. 6). In der Einzelbetrachtung liegt die Adhärenz der Friseur:innen bei den allgemeinen Schutzmaßnahmen signifikant und mit moderater Effektstärke unter der der beiden anderen Subindizes.

**Table 6:** 

	MW	SD	Median	Spanne	*N*_(gültig)_
*Gesamtindex*	0,75	0,14	0,77	0,40–1,00	162
*Subindizes*^*a*^					
1. Allgemeine Schutzmaßnahmen	0,68	0,21	0,67	0,13–1,00	162
2. Schutzmaßnahmen im Kontakt mit dem/der Beobachter:in	0,77	0,19	0,80	0,20–1,00	162
3. Persönliche Schutzmaßnahmen der Friseur:innen	0,79	0,19	0,67	0,25–1,00	162

Datenbasis: *n* = 162

Möglicher Wertebereich von 0–1 (keine bis bestmögliche SARS-CoV-2-Arbeitsschutzstandard-Adhärenz)

Post-hoc-Unterschiedstests zwischen den Subindizes (nonparametrischer Wilcoxon-Test): Index 1 vs. 2 und 3 jeweils *p* = 0,000 (Effektstärke r = 0,33 bzw. 0,38)

*MW* Mittelwert, *SD* Standardabweichung

^a^Siehe jeweils Tab. 1, 2 und 3

Statistische Unterschiede in den 3 Städten wurden weder auf der Ebene der einzelnen Beobachtungen noch in der standardisierten Gesamtschau gefunden.

## Diskussion

Die vorgestellte Studie beschäftigt sich mit der Adhärenz von Friseur:innen, den in einem branchenspezifischen Arbeitsschutzstandard festgehaltenen Empfehlungen zur Infektionsvermeidung während der SARS-CoV-2-Pandemie zu folgen.

Passend zu den bisherigen Erfahrungen der BGW-Präventionsdienste hinsichtlich der Arbeitsschutzadhärenz bei eher bekannten Standards (geschätzt zwei Drittel) ist der Fremd- und Selbstschutz in der SARS-CoV‑2 Pandemie – insbesondere hinsichtlich des individuellen Verhaltens – insgesamt als relativ gut zu bewerten, wenn auch noch mit etwas *Luft nach oben*.

Unsere Resultate fügen sich zum einen ein in die Ergebnisse einer Vor-Ort-Analyse von Arbeitsschutzexpert:innen in verschiedenen Branchen u. a. zum Umsetzungsgrad von SARS-CoV-2-Arbeits- und Infektionsschutzmaßnahmen [14]. Sechs Prozent berichteten, dass sich nach Auskunft der besuchten Friseur:innen 64 % der Beschäftigten vollständig bzw. überwiegend an die betrieblich vorgegebenen Verhaltensregeln halten. 56 % der 724 online Befragten berichteten, dass von betrieblichen Entscheidungsträgern die Schriften des zuständigen Unfallversicherungsträgers zum Umgang mit der SARS-CoV-2-Pandemie als Informationsquelle genutzt und mehrheitlich auch für verständlich, praxistauglich und wirksam gehalten wurden.

Zum anderen fügen sich unsere Ergebnisse recht gut in die von (unveröffentlichten) standardisierten Befragungen der BGW-Präventionsdienste ermittelten Ergebnisse ein. Die BGW-Präventionsdienste besuchten im vergangenen Jahr stichprobenartig rund 400 Friseursalons und überprüften den Zielerreichungsgrad von insgesamt 17 Maßnahmen zum Arbeitsschutz. Diese umfassten neben der Befolgung von Empfehlungen zur Arbeitsplatzgestaltung und zum persönlichen Infektionsschutz auch die Durchführung von Gefährdungsbeurteilungen, Unterweisungen und arbeitsmedizinischer Vorsorge. Die zu erreichenden Ziele wurden insgesamt zu über 90 % in den besuchten Friseursalons bestätigt [15].

Die Präventionsdienste können die Gewissenhaftigkeit bei der Beantwortung des persönlichen Infektionsschutzverhaltens bei ihrem überprüfenden und beratenden Besuch nicht kontrollieren. Grundsätzlich sind daher auch Antworten im Sinne sozialer Erwünschtheit zu erwarten. Deshalb ist es erfreulich, dass die Beurteilung der Arbeitsschutzstandard-Adhärenz in den Beobachtungen von Kund:innen nicht maßgeblich von denen der Präventionsdienste abweicht.

Auch könnten unsere Ergebnisse zur SARS-CoV-2-Arbeitsschutzstandard-Adhärenz noch eine Unterschätzung darstellen. In einer individuellen Situation kann der Arbeits- und Infektionsschutz auch gewährleistet sein, wenn z. B. keine Bodenmarkierungen oder Schutzschilder implementiert wurden.

Die Diskrepanz der Ergebnisse zu früheren, wenn auch nichtsystematisch erhobenen negativen Erfahrungen zur Befolgung von Standards lässt sich sicher nicht zuletzt mit dem allgemeinen hohen Bedrohungsgefühl in der Bevölkerung durch SARS-CoV‑2 und der Sorge um wirtschaftliche Nöte bei längeren Betriebsschließungen im Lockdown erklären. Es ist zu vermuten, dass nicht zuletzt auch eine breite Abstimmung mit Vertreter:innen aus dem Friseurhandwerk und eine ausgeprägte FAQ-Sammlung auf den Seiten der BGW zu den Empfehlungen zu einem guten Ergebnis führten.

In mehr als der Hälfte der Friseursalons wurde trotz kalter Herbst- und Wintermonate auf die eine oder andere Art zusätzlich in Form von Dauer- oder Stoßlüftung gelüftet. Neueste Erkenntnisse zur Luftübertragbarkeit von SARS-CoV‑2 belegen die Bedeutsamkeit konsequenten Lüftens; diesem Umstand wurde auch in der Aktualisierung des Arbeitsschutzstandards Rechnung getragen [16, 17].

Erfreulich ist das mehrheitlich konsequente Tragen von Mund-Nasen-Bedeckungen während der Arbeit. Dass dies Infektionen auch im Friseursalon verhindert, wurde eindrücklich durch eine amerikanische Studie mit zwei symptomatischen Friseur:innen mit bestätigter COVID-19-Erkrankung belegt, bei denen alle 139 ebenfalls gesichtsmaskentragenden Kund:innen nicht infiziert wurden [18]. Welchen Anteil Friseursalons an einer Ausbreitung von COVID-19-Infektionen in der Bevölkerung tatsächlich haben, ist empirisch bislang nicht bekannt und soll erstmals in Belgien geklärt werden [19]. Einem Statement der britischen *Scientific Advisory Group for Emergencies* (SAGE) zufolge, scheint die SARS-CoV-2-Reproduktionsrate von 0,05 allerdings unerheblich, und auch die Krankenkassenstatistiken weisen eine vergleichsweise geringere Inzidenz auf, verglichen mit der Gesamtinzidenz der versicherten Beschäftigten (mündliche Mitteilung des BKK-Bundesverbandes vom 09.02.2021).

### Limitationen.

Angesichts der nichtsystematisch rekrutierten Gelegenheitsstichprobe kann eine Zugangsverzerrung nicht ausgeschlossen werden. Dies lassen neben persönlichen Informationen auch vereinzelte Pressemeldungen vermuten [20, 21]. Die Lüftungsqualität kann durch die Beobachtungen von Kund:innen nicht prima vista beurteilt werden. Hierzu wären weitere technische/fachliche Beurteilungen erforderlich. Inwieweit der Arbeitsschutzstandard zur Bewältigung des Infektionsrisikos durch SARS-CoV‑2 in Friseursalons selbst bekannt ist, haben wir mit unserem Studienansatz nicht erfassen können.

## Ausblick

Möglicherweise werden andere Arbeitsschutzregeln und -empfehlungen mit geringerer aktueller Brisanz als der hier untersuchten in geringerem Maß umgesetzt. Dies sollte weiter untersucht und daraus ggf. Schlüsse für die weitere Betriebsberatung durch die berufsgenossenschaftlichen Präventionsdienste gezogen werden.

## Fazit für die Praxis

- Während verdeckter teilnehmender Beobachtungen von Friseurkund:innen Ende 2020 wurden die im branchenspezifischen BGW-Arbeitsschutzstandard empfohlenen SARS-CoV-2-Schutzmaßnahmen mehrheitlich umgesetzt.
- Dies gilt insbesondere für individuelle Schutzmaßnahmen von Friseur:innen sowie deren Kund:innen.
- Die Adhärenz anderer – weniger bekannter – Arbeitsschutzregelungen sollte in verschiedenen Branchen weiter untersucht werden.
- Ziel wäre die Beantwortung der Frage, an welchen Stellen Betriebe (weitere) konkrete Unterstützung bei der Umsetzung von Arbeitsschutzmaßnahmen benötigen.
